# Optic Nerve Head and Retinal Abnormalities Associated with Congenital Fibrosis of the Extraocular Muscles

**DOI:** 10.3390/ijms22052575

**Published:** 2021-03-04

**Authors:** Mervyn G. Thomas, Gail D. E. Maconachie, Helen J. Kuht, Wai-Man Chan, Viral Sheth, Michael Hisaund, Rebecca J. McLean, Brenda Barry, Bashir Al-Diri, Frank A. Proudlock, Zhanhan Tu, Elizabeth C. Engle, Irene Gottlob

**Affiliations:** 1 The University of Leicester Ulverscroft Eye Unit, Department of Neuroscience, Psychology and Behaviour, University of Leicester, RKCSB, PO Box 65, Leicester LE2 7LX, UK; g.d.maconachie@sheffield.ac.uk (G.D.E.M.); hjk15@le.ac.uk (H.J.K.); vs109@le.ac.uk (V.S.); mh486@le.ac.uk (M.H.); rjm19@le.ac.uk (R.J.M.); fap1@le.ac.uk (F.A.P.); zt33@le.ac.uk (Z.T.); 2 Division of Ophthalmology & Orthoptics, Health Sciences School, University of Sheffield, Sheffield S10 2TN, UK; 3 Department of Neurology, Boston Children’s Hospital, Boston, MA 02115, USA; wchan@enders.tch.harvard.edu (W.-M.C.); Brenda.Barry2@childrens.harvard.edu (B.B.); elizabeth.engle@childrens.harvard.edu (E.C.E.); 4 Howard Hughes Medical Institute, Chevy Chase, Maryland, MD 20815, USA; 5 Brayford Pool Campus, School of Computer Science, University of Lincoln, Lincoln LN6 7TS, UK; baldiri@lincoln.ac.uk; 6 Departments of Neurology and Ophthalmology, Boston Children’s Hospital, Boston, MA 02115, USA; 7 Departments of Neurology and Ophthalmology, Harvard Medical School, Boston, MA 02115, USA; 8 Broad Institute of Harvard and MIT, Cambridge, MA 02142, USA

**Keywords:** congenital fibrosis of extraocular muscles, optic nerve hypoplasia, optical coherence tomography, retinal ganglion cells, development, congenital cranial dysinnervation disorders

## Abstract

Congenital fibrosis of the extraocular muscles (CFEOM) is a congenital cranial dysinnervation disorder caused by developmental abnormalities affecting cranial nerves/nuclei innervating the extraocular muscles. Autosomal dominant CFEOM arises from heterozygous missense mutations of *KIF21A* or *TUBB3*. Although spatiotemporal expression studies have shown KIF21A and TUBB3 expression in developing retinal ganglion cells, it is unclear whether dysinnervation extends beyond the oculomotor system. We aimed to investigate whether dysinnervation extends to the visual system by performing high-resolution optical coherence tomography (OCT) scans characterizing retinal ganglion cells within the optic nerve head and retina. Sixteen patients with CFEOM were screened for mutations in *KIF21A*, *TUBB3*, and *TUBB2B*. Six patients had apparent optic nerve hypoplasia. OCT showed neuro-retinal rim loss. Disc diameter, rim width, rim area, and peripapillary nerve fiber layer thickness were significantly reduced in CFEOM patients compared to controls (*p* < 0.005). Situs inversus of retinal vessels was seen in five patients. Our study provides evidence of structural optic nerve and retinal changes in CFEOM. We show for the first time that there are widespread retinal changes beyond the retinal ganglion cells in patients with CFEOM. This study shows that the phenotype in CFEOM extends beyond the motor nerves.

## 1. Introduction

Congenital fibrosis of the extraocular muscles (CFEOM) is a congenital cranial dysinnervation disorder characterized by non-progressive ophthalmoplegia with or without blepharoptosis [1]. CFEOM is genetically heterogeneous and can be further classified into several subtypes, namely CFEOM1A, CFEOM1B, CFEOM2, CFEOM3A, CFEOM3B, CFEOM3C, Tukel syndrome, and CFEOM3 with polymicrogyria [2]. The minimum prevalence of this disorder was found to be 1:230,000 [3]. CFEOM1 is the most common subtype and typically presents with bilateral congenital non-progressive restrictive ophthalmoplegia with the eyes partially or fully fixed in depression, limited upgaze, and blepharoptosis [3]. CFEOM1 is inherited in an autosomal dominant manner and can arise from mutations of *KIF21A* (CFEOM1A) [4] or *TUBB3* (CFEOM1B) [5]. CFEOM2 is characterized by congenital non-progressive bilateral exotropic ophthalmoplegia and blepharoptosis. Vertical and horizontal eye movements are severely restricted, and sluggish pupillary reactions to light have been described [6]. CFEOM2 is inherited in an autosomal recessive manner and arises from *PHOX2A* mutations [7]. CFEOM3 has variable clinical features, which can include varying degrees of congenital non-progressive external ophthalmoplegia and blepharoptosis. The severity can range from mild to complete ophthalmoplegia and can be unilateral or bilateral [5]. There can be significant overlap of phenotypical features with CFEOM1 or CFEOM2, and thus they can be clinically indistinguishable. CFEOM3 is inherited in an autosomal dominant manner and arises from *TUBB3* or *TUBB2B* mutations [5]. Recently we have reported that congenital monocular elevation deficiency, or double elevator palsy, can be part of CFEOM3 and arise from *TUBB3* mutations [8].

Magnetic resonance imaging (MRI) studies in CFEOM secondary to *KIF21A* and *TUBB3* mutations have provided evidence for widespread orbital dysinnervation, which can include optic nerve hypoplasia [9,10]. Clinical examination aided by fundus photos have also been used to describe disc excavation and optic nerve hypoplasia in CFEOM1 and CFEOM3; however, sequence analysis only identified one patient with a *KIF21A* mutation [11,12]. Retinal dysfunction has been identified in CFEOM2 with delayed or depressed rod and cone responses detected on electroretinogram (ERG) [13]. These studies suggest abnormalities in CFEOM extend beyond motor nerves and can involve the optic nerve and retina as well. Spectral domain optical coherence tomography (SD-OCT) provides an opportunity to perform non-invasive high-resolution in vivo imaging of the retina and optic nerve head. However, to date, there have been no studies using OCT to investigate the retinal or optic nerve head morphology in CFEOM. The advent of handheld ultra-high-resolution SD-OCT enables detailed characterization of the retina and optic nerve head morphology in pediatric populations and patients who find it difficult to use a table-mounted OCT device due to abnormal head posture or large angle strabismus [14]. In this study, we aimed to genotype a cohort of patients with CFEOM and also to utilize a handheld SD-OCT to phenotypically characterize the optic nerve head and foveal morphology in patients with CFEOM.

## 2. Results

### 2.1. Clinical and Genetic Characteristics

**Figure 1:**
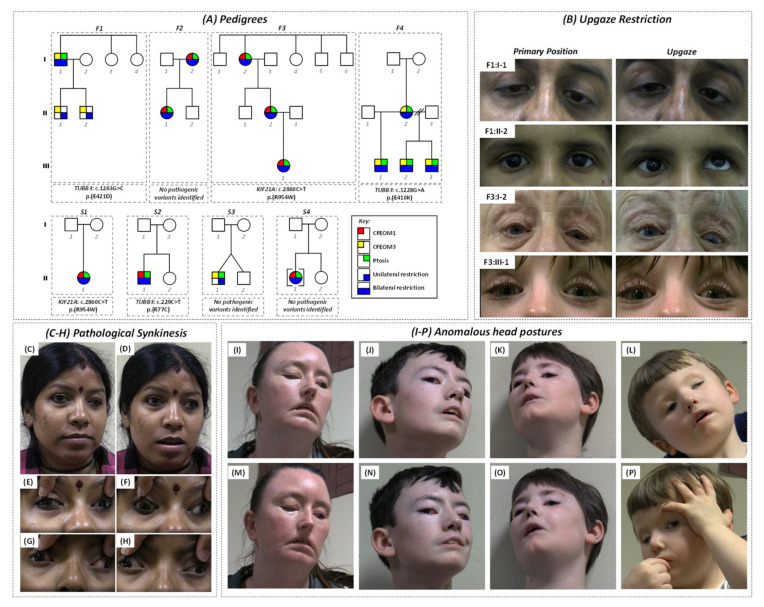
(**A**) Pedigrees of families (F1–F4) and singletons (S1–S4) diagnosed with congenital fibrosis of the extraocular muscles. Genotypes are shown below the pedigrees. (**B**) Eye position in subjects with congenital fibrosis of extraocular muscles when observing a target located centrally (primary position) and superiorly (upgaze). Subject F1:I-1 has bilateral blepharoptosis, left esotropia, and no elevation in both eyes. However, his son (subject F1:II-3) has CFEOM3 phenotype limited to the right eye; he is unable to elevate the eye above the midline. There was no blepharoptosis and ductions were normal for the left eye. Subject F3:I-2 and subject S1:II-1 exhibited a CFEOM1 phenotype with bilateral blepharoptosis and inability to elevate either eye. Subject F3:I-2 also had a left exotropia and hypotropia. (**C**–**H**) Congenital pathologic synkinesis showing Marcus Gunn jaw winking phenomenon and synergistic divergence. Subject F2:I-2 had bilateral blepharoptosis, face turn to the left, and left exotropia with hypotropia (**C**). Retraction of the left upper eyelid is observed with mouth opening (**D**). Eye positions on central fixation (**E**) and downgaze (**F**) are shown. Synkinetic abduction of the left eye is observed on infraversion. Similar to F2:II-1, there is synkinetic abduction from central fixation (**G**) to downgaze (**H**). (**I**–**P**) Abnormal head postures observed when fixing at a target centrally in family F4 (**I**–**L**). Subjects F4:I-2 (**I**) and F4:II-1 (**J**) exhibit chin up and face turn to the left. Subject F4:II-2 has a head turn to the right with chin up position (**K**). Subject F4:II-3 has head tilt to the right with chin up position (**L**). All subjects had large angle exotropia. Facial weakness is demonstrated in all subjects when asked to smile (**M**–**O**). Drooping of the corner of the mouth and flattening of forehead and nasolabial folds are seen. Subject F4:II-3 had severe blepharoptosis of the left eye and frequently raised his upper left eyelid and brow using his finger in order to fixate with the left eye (**P**).

The pedigrees and characteristics of individuals diagnosed with CFEOM are shown in Figure 1A and Supplementary Table S1, respectively. The phenotypes and genotypes associated with families F1 [8] and F4 [15] and subject S1:II-1 [4,16] have previously been described.

Affected subjects in F1 had significant intra-familial clinical variability and each harbored a heterozygous *TUBB3* mutation (c.1263G > C, p.Glu421Asp) [8]. Subject F1:I-1 had bilateral blepharoptosis, left esotropia, and bilateral restriction on elevation (Figure 1B); however, both his children (F1:II-1 and F1:II-2) had unilateral restriction without ptosis (Figure 1B) [8].

Both subjects in family F2 had bilateral blepharoptosis and restrictive ophthalmoplegia. Subject F2:I-2 had asymmetrical ptosis, Marcus Gunn jaw winking phenomenon, and synergistic divergence (Figure 1C–F). The daughter (subject F2:II-1) had synergistic divergence (Figure 1G,H). Mutation screening was unrevealing.

Affected members of family F3 had bilateral blepharoptosis and restrictive ophthalmoplegia consistent with CFEOM1 (Figure 1B), and each had a heterozygous *KIF21A* mutation (c.2860C > T, p.(Arg954Trp)—Supplementary Figure S1).

Affected members of family F4 had bilateral blepharoptosis, severe restrictive ophthalmoplegia, facial weakness, anosmia, and intellectual disability; the mother (F4:I-2) harbored a de novo heterozygous *TUBB3* mutation (c.1228G > A, p.(Glu410Lys)), which then segregated with the phenotype in all three of her children [15].

Singletons S1:II-1, S2:II-1, and S4:II-1 had bilateral ptosis with limited supraduction. S1:II-1 had a de novo heterozygous *KIF21A* mutation (c.2860C > T, p.(Arg954Trp)) and, as a child, had right hypertropia observed during tooth brushing [4,16]. Sequence analysis in singleton S2:II-1 revealed a rare variant in *TUBB3* (c.229C > T, p.(Arg77Cys)) inherited from his unaffected mother (Supplementary Figure S1). This variant was absent in his unaffected father and sister. It is located within the GTPase domain and, based on in silico analysis, is predicted to be disease-causing by MutationTaster and benign by PolyPhen-2 (Supplementary Table S2). This residue is moderately conserved with a GERP score of 4.57. It is absent from dbSNP and gnomAD, although R77H is in gnomAD once in the heterozygous state (3.98 × 10^−6^). Overall, based on ACMG criteria, this was classified as a variant of unknown significance. S3:II-1 had unilateral ptosis with limited vertical gaze, and both singletons S3 and S4 remain genetically unsolved with no mutations in *KIF21A*, *PHOX2A*, *TUBB3*, or *TUBB2B*. There was no family history of strabismus in S3 or S4.

Subject F1:I-1 had been diagnosed with primary open-angle glaucoma and was subsequently excluded from the optic nerve and foveal analysis. All other subjects had normal intraocular pressures and no other ocular co-morbidities. Confrontational visual fields were grossly normal, and cooperation was poor in F4:II-1, F4:II-2, and F4:III-3. There was no relative afferent pupillary defect in any of the patients.

The mean BCVA were 0.36 ± 0.09 logMAR (right eye) and 0.56 ± 0.13 logMAR (left eye) (mean ± SEM). Eleven subjects were diagnosed with amblyopia with a mean interocular visual acuity difference of 0.36 logMAR (range 0.2–0.8 logMAR). The mean BCVA (excluding amblyopic eyes) in the CFEOM subjects was 0.31 ± 0.10 logMAR (mean ± SEM). This suggests that there was subnormal vision even after accounting for amblyopia.

### 2.2. Optic Nerve Abnormalities

**Figure 2:**
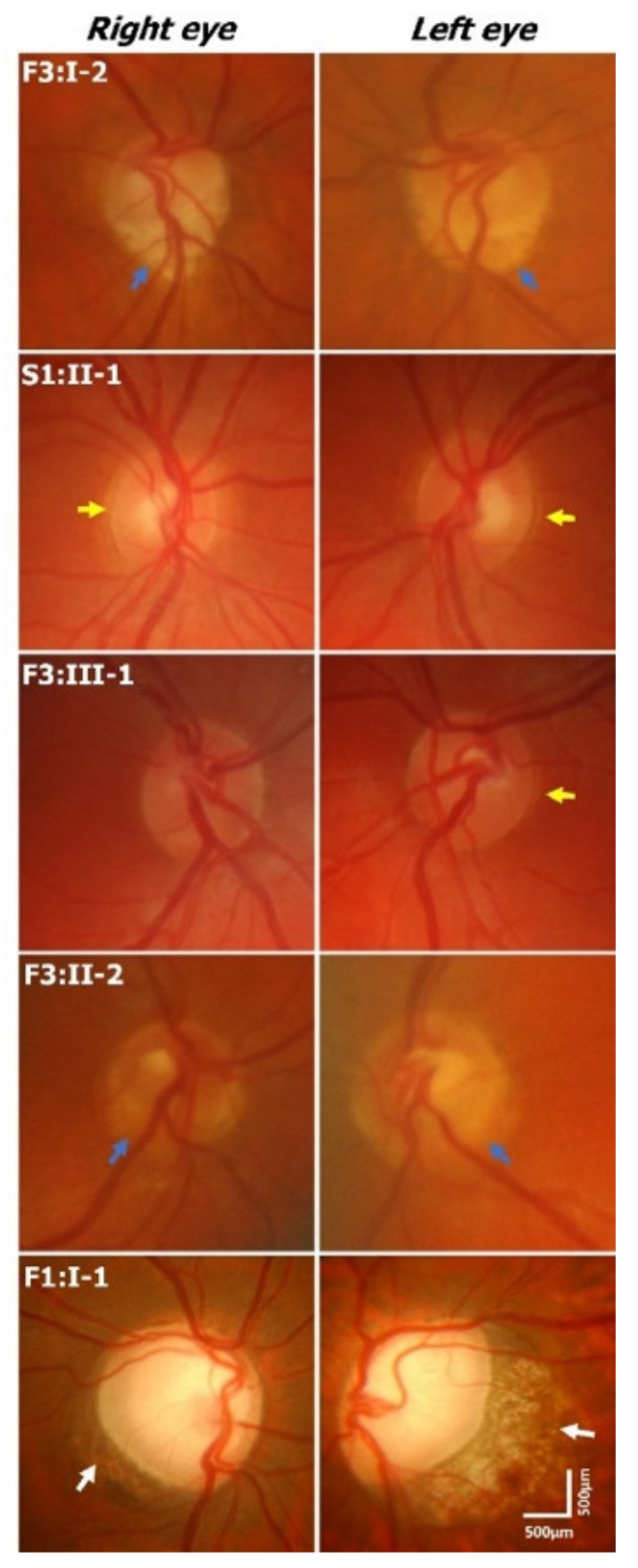
Fundus photos of subjects with CFEOM. In subject F3:1-2, the disc appears tilted with a small cup and inferior yellowish mottled halo (blue arrows). There is situs inversus of the blood vessels. Subject S1:II-1 has a faint halo surrounding the disc, which is a subtle incomplete double ring (yellow arrows). This is also seen in the left eye of subject F3:III-1, with an abnormal course of retinal vessels seen in both eyes. Subject F3:II-2 has hypoplastic optic nerves with significant inferior peripapillary scleral halos (blue arrows). Abnormal course of retinal vessels is seen. Subject F1:I-1 has tilted disc with myopic crescent (white arrow) and significant disc excavation and pallor. Scale bar represents 500 microns.

**Figure 3:**
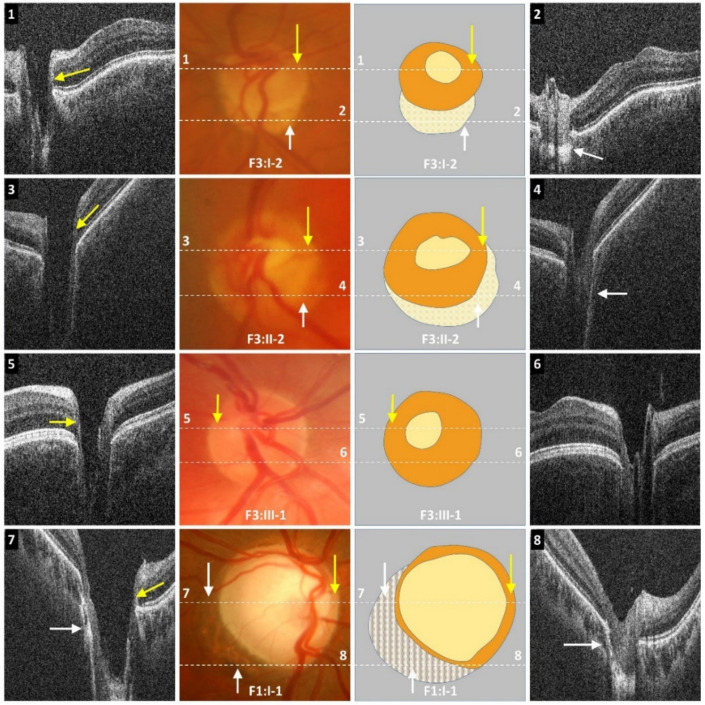
Optical coherence tomograms with corresponding disc photos. Dotted white line represents location of the OCT B-scan overlaid on the disc photo. Schematic shows borders of disc (orange) with the cup centrally (yellow), double ring (pale yellow), and peripapillary atrophy (hatched). Partial double ring/atrophy (F3:I-2 and F3:II-2) is seen below disc border (white arrows). In subject F3:I-2, the first scan (**1**) shows thin temporal neuro-retinal rim (yellow arrow); this is not evident on the disc photo as the borders of the cup are not clear and the cup appears significantly smaller. The location of the neuro-retinal rim on the fundus photo is highlighted with the yellow arrow. The second scan (**2**) is through a region of yellow mottled appearance. The OCT shows a hyper-reflective region posteriorly (white arrow). Disc photo of subject F3:II-2 shows appearance of a small cup. However, on OCT (**3**) the rim is very thin (yellow arrow), and the cup is deep and appears larger than on the fundus photo. The OCT through the inferior aspect of the disc (**4**) shows an optic cup that is not evident on the fundus photo. Disc photo in F3:III-1 shows a small cup with large neuro-retinal rims. However, OCT (**5**) shows thin neuro-retinal rim temporally (yellow arrow). OCT (**6**) below the inferior border of the presumed cup (from the fundus photo and illustration) shows a well-formed cup, although not as deep as the OCT through the center of the cup (**5**). In subject F1:I-1, this abnormal sloping is evident in the region of peripapillary atrophy (white arrow) in both scans (**7**,**8**). There is also a thin neuro-retinal rim with good correlation between disc photos and the OCT (yellow arrow).

Optic disc examination revealed features such as peripapillary atrophy and double-ring sign (see examples in Figure 2 and Supplementary Table S1). Partial double-ring sign or atrophy was seen in 6/16 (38%) patients (Figure 2 and Figure 3). Tilted discs were seen in 2/16 (13%) patients. Interestingly, fundus photos showed healthy neuro-retinal rims with small cups in six patients; however, the OCT revealed large excavated cups, thus giving a pseudo-normal appearance on fundus examination (Figure 3). Subject F3:II-2 has a yellow central area mimicking the cup in fundus photos; however, OCT shows a deep cup with thin neuro-retinal rims. There was a significant difference between the cup/disc ratio based on fundus photos/clinical examination and OCT. The cup/disc ratio based on fundus photos (mean ± SEM = 0.42 ± 0.06) was significantly smaller (*p* = 0.0003) when compared to OCT measurements (mean ± SEM = 0.76 ± 0.03). In all cases, the cup/disc ratio was underestimated on fundus photos/clinical examination. OCT through the double ring shows loss of RPE with hyper-reflectivity posterior to Bruch’s membrane (scan 2 in Figure 3). Similarly, in peripapillary atrophy we find abnormal sloping of the RPE and hyper-reflectivity posterior to the RPE (scan 8 in Figure 3).

Reliability analyses between examiners revealed high intraclass correlation coefficients for disc diameter (0.93; 95% CI = 0.86–0.96), rim width (0.90; 95% CI = 0.80–0.95), cup diameter (0.96; 95% CI = 0.93–0.98), and cup depth (0.96; 95% CI = 0.92–0.98).

**Figure 4:**
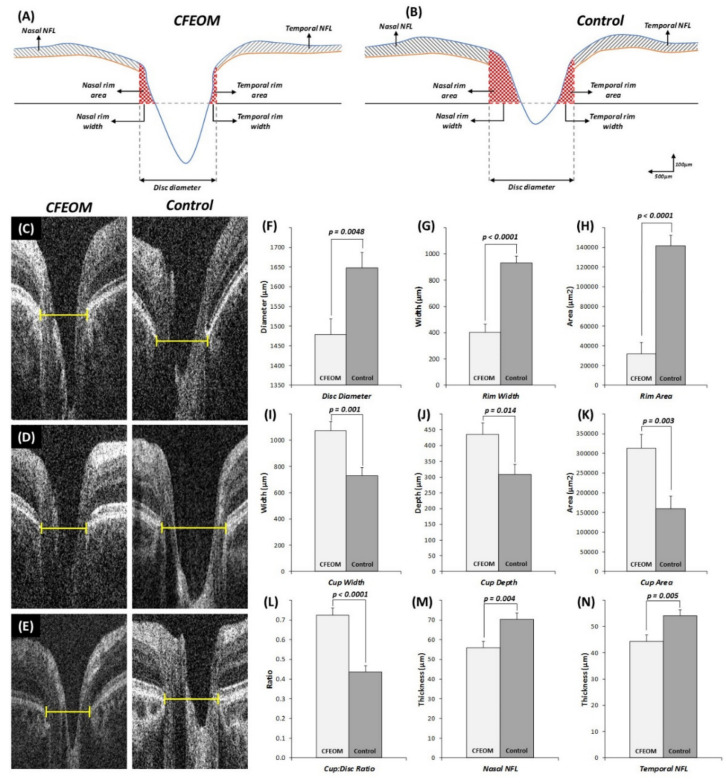
Schematic representation based on average measurements of disc parameters in CFEOM (**A**) and controls (**B**). Examples of disc optical coherence tomograms in CFEOM patients and controls (**C**–**E**). Yellow lines represent the horizontal disc diameters measured from the termination of the retinal pigment epithelium. Graphs show mean and error bars representing the standard error of mean for disc diameter (**F**), rim width (**G**), rim area (**H**), cup width (**I**), cup depth (**J**), cup area (**K**), cup/disc ratio (**L**), nasal nerve fiber layer (NFL), thickness (**M**), and temporal NFL thickness (**N**). *p* values are shown above error bars.

Quantitative analysis showed that the horizontal disc diameter width was significantly reduced in CFEOM patients compared to controls (Figure 4F, mean difference ± standard error of mean (MD ± SEM) = 168.8 ± 55.9 µm, *p* = 0.0048). Rim width (Figure 4G, MD ± SEM = 527.5 ± 80.3 µm, *p* < 0.0001) and total rim area (Figure 4H, MD ± SEM = 109872.4 ± 15264.4 µm^2^, *p* < 0.0001) were significantly reduced in CFEOM. Cup width (Figure 4I, MD ± SEM = 346.2 ± 92.9 µm *p* = 0.001), cup depth (Figure 4J, MD ± SEM = 127.3 ± 48.3 µm, *p* = 0.014), and cup area (Figure 4K, MD ± SEM = 153535.2 ± 46908.8 µm^2^, *p* = 0.003) were significantly increased in CFEOM compared to controls. Cup/disc ratio was significantly larger in CFEOM compared to controls (Figure 4L, MD ± SEM = 0.29 ± 0.05, *p* < 0.0001). RNFL thickness was significantly reduced both nasally (Figure 4M, MD ± SEM = 14.4 ± 4.5 µm, *p* = 0.004) and temporally (Figure 4N, MD ± SEM = 9.7 ± 3.2 µm, *p* = 0.005).

No significant correlation was seen between visual acuity and optic nerve head parameters (disc diameter, rim width, rim area, and RNFL thickness) (*p* > 0.05).

### 2.3. Retinal Vascular Abnormalities

We obtained fundus photos in 13 patients. In 4/13 patients the central retinal vessels emerge off-center or from the peripheral part of the optic nerve, and the temporal vessels course nasally before turning temporally—features consistent with a situs inversus pattern (Figure 2 and Supplementary Table S1).

No significant differences were noted in the branching geometry at the bifurcation (angle θ, θ1, and θ2), diameter ratios (α, β, and λ), or junction exponent (K).

### 2.4. Foveal and Parafoveal Abnormalities

**Figure 5:**
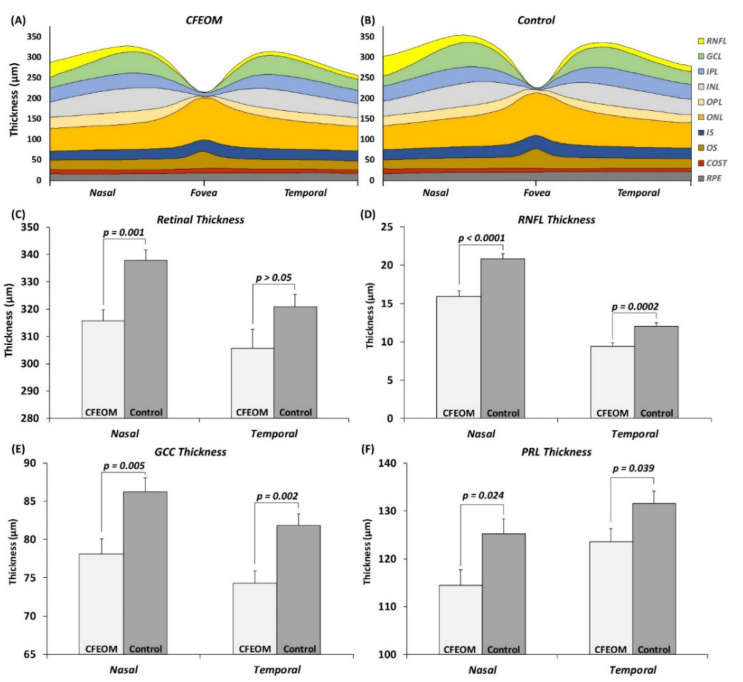
Schematic representation of average thickness of each retinal layer in CFEOM (**A**) and controls (**B**). Significant differences were observed for nasal retinal thickness (**C**), retinal nerve fiber layer (RNFL) (**D**), ganglion cell complex (GCC) (**E**), and photoreceptor layer (PRL) thickness (**F**). Bar charts represent mean and standard error (µm). *p* values are shown above each bar. Abbreviations: RNFL = retinal nerve fiber layer, GCL = ganglion cell layer, IPL = inner plexiform layer, INL = inner nuclear layer, OPL = outer plexiform layer, ONL = outer nuclear layer, IS = inner segment, OS = outer segment, COST = cone outer segment tip, and RPE = retinal pigment epithelium.

Visual inspection of the tomograms did not reveal any gross foveal abnormalities. However quantitative analysis showed significant differences in the parafoveal region. Nasal parafoveal retinal thickness was significantly reduced in CFEOM compared to controls (Figure 5C, MD ± SEM = 22.0 ± 5.6 µm, *p* = 0.001). This was due to a significantly thinner RNFL (Figure 5D, MD ± SEM = 4.8 ± 0.9 µm, *p* < 0.0001), GCC (Figure 5E, MD ± SEM = 8.1 ± 2.7 µm, *p* = 0.005), and PRL (Figure 5F, MD ± SEM = 10.8 ± 4.5 µm, *p* = 0.024). Although the temporal parafoveal retinal thickness was not significantly different in CFEOM (*p* > 0.05), the RNFL (Figure 5D, MD ± SEM = 2.6 ± 0.6 µm, *p* = 0.0002), GCC (Figure 5E, MD ± SEM = 7.5 ± 2.2 µm, *p* = 0.002), and PRL (Figure 5F, MD ± SEM = 7.9 ± 3.7 µm, *p* = 0.039) were significantly thinner in CFEOM compared to controls.

## 3. Discussion

This is the first study showing widespread retinal and optic nerve head changes in patients with CFEOM. We identify that patients with CFEOM have smaller optic nerves with deeper optic cups and significantly thinner neuro-retinal rims. The reduced visual acuity in CFEOM could be partly attributed to the retinal and optic nerve changes seen. However, likely due to the presence of amblyopia, we did not observe a correlation with visual acuity. Significant retinal vascular abnormalities were seen in 4/13 patients.

The exact pathogenesis of optic nerve hypoplasia in patients with CFEOM is unclear. However, using MRI studies, optic nerve hypoplasia has previously been described in patients with *KIF21A* mutations [10] and *TUBB3* mutations [9]. In CFEOM1, there is approximately between 30% and 40% reduction in optic nerve cross-sectional area on MRI [10]. Similarly, in CFEOM3, there is approximately 35% reduction in optic nerve cross-sectional area [9]. In this study, we find on average approximately 10% reduction in horizontal disc diameter. The differences between the studies are likely due to differences in (a) imaging modalities (MRI versus SD-OCT), (b) type of measurements (cross-sectional area versus horizontal disc diameter), and (c) location of optic nerve measurements (2 mm from globe versus scleral opening).

On ophthalmoscopy, we could identify features of optic nerve hypoplasia, such as an incomplete double-ring sign, in 6/16 (38%) patients. This was seen in both patients with *KIF21A* and *TUBB3* mutations (Supplementary Table S1). In both CFEOM1 and CFEOM3, optic nerve hypoplasia with double-ring sign has been described [9,10,12]. In 2/10 (20%) patients with CFEOM3, a double-ring sign has been described; both subjects had TUBB3 variants resulting in amino acid substitution R262C [12]. Khan et al. identified a higher rate of optic nerve hypoplasia (5/10 (50%)) in CFEOM in comparison to our study and previous work. The genetic diagnosis was only identified in 1/10 patients. Thus, the difference could arise due to the difference in study populations, since most families studied by Khan et al. were from consanguineous families [12].

In this study, we show that the GCC in patients with CFEOM is thinner compared to controls. This could potentially be the basis for smaller optic nerves seen in patients with CFEOM. Within the retina, KIF21A expression was observed in the retinal ganglion cell bodies [17]. However, the Kif21a R954W knock-in mouse model did not demonstrate any abnormalities in retinal ganglion cell axonal projections [18]. This suggests that pathogenesis for the retinal phenotype observed in humans could be different from the Kif21aKI/KI model, since the R954W variant in humans can be associated with variable expressivity of optic nerve hypoplasia as described in this study and others [10,12].

TUBB3 has widespread expression within the developing neural retina. It is expressed in the retinal ganglion cells, amacrine cells, horizontal cell processes, and cone photoreceptors [19]. In Tubb3-/- mice, there is a decrease in growth cone microtubule dynamics and a decreased neurite outgrowth rate in peripheral axons but no developmental pathology [20]. However, the roles within retinal cell populations have not been investigated. Based on our OCT findings and previous MRI work [9], it is possible that TUBB3 has a role in retinal neurogenesis. However, this requires further study. Optic nerve hypoplasia has been reported in four subjects from two consanguineous families with *TUBA8* mutations and polymicrogyria [21]. Similarly, optic nerve hypoplasia has been reported with *TUBA1A* mutations [22], thus suggesting that tubulin gene mutations in general can cause optic nerve hypoplasia [9,21,22].

We found a significantly reduced PRL in patients with CFEOM when compared to controls. TUBB3 is expressed within the developing cone photoreceptors [19]; however, there is no direct evidence of KIF21A involvement in photoreceptor development. Photoreceptor changes in CFEOM are a common but perhaps poorly explored manifestation of this disorder. With the advent of handheld SD-OCT, it will be possible to document these structural changes in a larger cohort of patients to identify specific genotype–phenotype correlations. The vascular abnormalities described in this study were only associated with *KIF21A* mutations. Kinesin proteins participate in intraflagellar transport to form cilia, which are important to direct nodal flow of extra-embryonic fluid, and thus affect the formation of the left–right axis during development [23]. Whether this process is perturbed due to *KIF21A* mutations resulting in situs inversus of retinal vessels requires further study.

In conclusion, we describe for the first time widespread optic nerve and retinal abnormalities in CFEOM that can be detected using SD-OCT. Changes are consistent with neuro-retinal expression patterns of KIF21A and TUBB3. OCT showed that loss of neuro-retinal rim was much more pronounced than apparent on fundus examination, where some of the patients had pseudo-normal discs. This expands the phenotypic spectrum associated with CFEOM and highlights the need for further research into the molecular mechanisms affecting development of the afferent pathways in this condition.

## 4. Materials and Methods

### 4.1. Subjects

Sixteen patients with CFEOM (8 male, 8 female; mean age ± standard deviation = 20 ± 16.80 years) from eight families and sixteen age-, gender-, and ethnicity-matched healthy controls participated in this study. All participants underwent a complete ophthalmic and orthoptic examination, including best-corrected visual acuity (BCVA), ocular motility, pupil examination, slit-lamp examination, refraction, intraocular pressure measurements, and dilated fundoscopy. Fundus photos were acquired using Zeiss VisucamPRO NM (Carl Zeiss Meditec Inc, Jena, Germany) or Pictor (Volk Optical Inc, Mentor, OH, USA). Intraocular pressure was measured using either Goldmann applanation tonometer or iCare rebound tonometer, TA01i (Tiolat Oy, Helsinki, Finland).

The study adhered to the tenets of the Declaration of Helsinki and was approved by the local ethics committee. Written informed consent was obtained from all participants or their parents or guardians.

### 4.2. Sequencing

Saliva samples (Oragene DNA sample Collection Kit (OG-500, DNA Genotek Inc., Ottawa, ON, Canada)) were obtained from all family members. DNA was extracted from the saliva samples. All coding exons and intron-exon boundaries of *TUBB3* and *TUBB2B* and exons 8, 20, and 21 of *KIF21A* were sequenced as previously reported [4,5,24]. Primer sequences are available on request.

### 4.3. Optical Coherence Tomography Acquisition

A handheld SD-OCT (Envisu C2300, Leica Microsystems, Wetzlar, Germany) was used for imaging the optic nerve head (ONH) and fovea. All images were obtained without sedation. The scan protocols used in this study have been previously described [25,26]. Scanning windows of 10 × 10 mm (A × B scans: 500 × 100) centered on the fovea and ONH separately was used to acquire them. We successfully obtained scans in 15/16 patients (93.7%).

### 4.4. Optic Nerve Head Analysis

A single B-scan through the deepest part of the optic cup was used for analysis as described elsewhere [25]. The scans were analyzed using ImageJ software version 1.48 [27]. The horizontal disc diameter was defined as the distance between the edges of Bruch’s membrane. The cup diameter was determined using a cup offset of 150 µm anterior to the disc axis. Cup depth was measured as the vertical distance from the cup base to the midpoint of the neuro-retinal peaks (Supplementary Figure S2 and Figure 4) [25]. The retinal nerve fiber layer (RNFL) thicknesses were calculated at 1.2 mm and 1.8 mm laterally from the ONH after adjusting for axial length, as described previously [14,25,26]. ONH analyses were performed independently by two authors (MT and MH) blinded to the diagnosis.

### 4.5. Foveal Analysis

The central foveal B-scan was selected based on the scan with the deepest foveal pit and features of cone photoreceptor specialization [28,29]. The retinal layers were segmented using a semi-automated ImageJ macro as described elsewhere [26]. Retinal thickness measurements obtained were the following: RNFL, ganglion cell complex (GCC), inner nuclear layer (INL), outer plexiform layer (OPL), photoreceptor layer (PRL) (sum of outer nuclear layer, inner segment, and outer segment), and retinal pigment epithelium (RPE).

At the fovea, we measured the foveal pit depth and pit diameter. For statistical analyses, we grouped the data as follows: (1) central foveal thickness measurements, (2) paracentral area (defined as average thickness of each layer 250 µm nasally to 250 µm temporally from the center), and (3) nasal and temporal areas (defined as average thickness of each layer 500 µm to 2000 µm from the center, nasally and temporally, respectively) (Supplementary Figure S3 and Figure 5).

### 4.6. Retinal Vessel Analysis

We utilized an automated algorithm for segmenting and measuring retinal vessels from fundus photos, by growing a “Ribbon of Twins” active contour model [30,31]. We calculated the number of bifurcations and different vessel tortuosity parameters. Features from each bifurcation (Supplementary Figure S4) were extracted. This included branching geometry at the bifurcation (angle θ, θ1, and θ2), diameter ratios (α, β, and λ), and junction exponent (**K**).

### 4.7. Statistical Analysis

Statistical analysis was performed using IBM SPSS Statistics software (version 24, IBM Corp.). A linear mixed model was used to determine significant differences in foveal, ONH, and vasculature measurements between CFEOM patients and controls. Within the model, eye (left vs. right) was assigned as a repeated measure, fixed factor was the diagnosis (CFEOM vs. controls), and random factors included age, gender, ethnicity, and refraction. Bonferroni correction was applied for multiple testing. *p* ≤ 0.05 was considered statistically significant. Intraclass correlation coefficients were calculated for disc width, rim width, cup width, and cup depth to assess reliability of measurements.

## Data Availability

We have deposited the variant data in Leiden Open Variation Database (LOVD) (available at: https://www.lovd.nl/3.0/home, accessed on 6 February 2021). The relevant individual accession IDs are as follows: #00327516, #00327518 - #00327528.

## Supplementary Materials

The following are available online at https://www.mdpi.com/1422-0067/22/5/2575/s1, Table S1: Genotype and clinical characteristics of patients with congenital fibrosis of extraocular muscles.
