# Supplementary material for: Optic Nerve Head and Retinal Abnormalities Associated with Congenital Fibrosis of the Extraocular Muscles
Source: Int J Mol Sci. 2021 Mar 4;22(5):2575. doi: 10.3390/ijms22052575 (PMC7961960; doi:10.3390/ijms22052575)
Supplement: Supplementary file 1 [file ijms-22-02575-s001.zip › supplementart S2+S3+S4.docx]

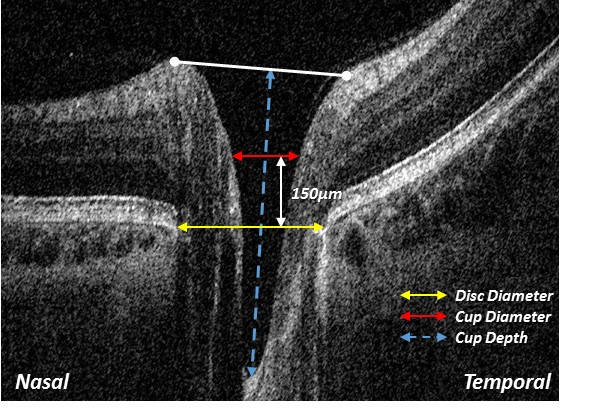


**Supplementary Figure 2.** Optic nerve head tomogram showing measurements obtained. Disc diameter (yellow arrow) was measured between the terminations of Bruch’s membrane. Cup diameter (red arrow) was obtained at an offset of 150µm from a line between the terminations of Bruch’s membrane. Cup depth (blue arrow) was the vertical distance between the cup base and the midpoint of the neuro-retinal peaks.


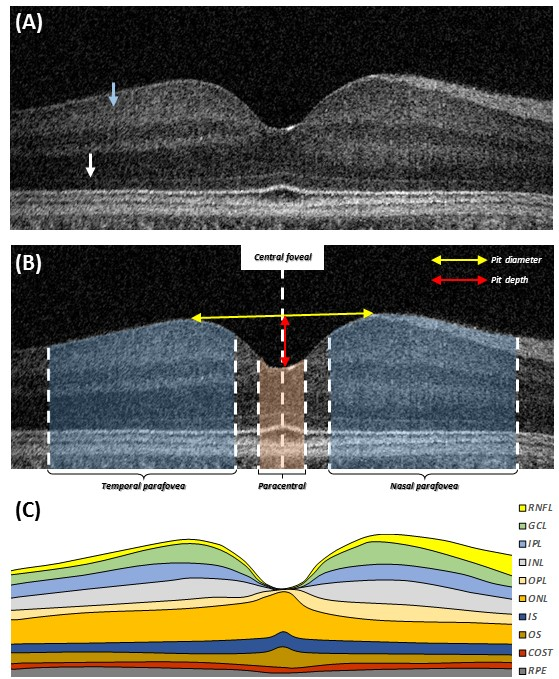


**Supplementary Figure 3.** Foveal tomogram and regions analyzed. In the parafoveal region distinguishing the ganglion cell layer and inner plexiform layer, interface can be difficult (blue arrow) (A). Similarly, the external limiting membrane can have a poor reflectivity in the parafoveal region, thus making segmentation difficult (white arrow) (A). Intra-retinal layer thickness measurements were obtained at the central fovea, paracentrally (250µm either side of the foveal centre), and at the parafovea (500µm to 2000µm from foveal center). Foveal pit diameter (yellow arrow) and pit depth (red arrow) were also measured (B). The different retinal layers segmented are shown (C). Abbreviations: RNFL = retinal nerve fiber layer, GCL = ganglion cell layer, IPL = inner plexiform layer, INL = inner nuclear layer, OPL = outer plexiform layer, ONL = outer nuclear layer, IS = inner segment, OS = outer segment, COST = cone outer segment tip, and RPE = retinal pigment epithelium.


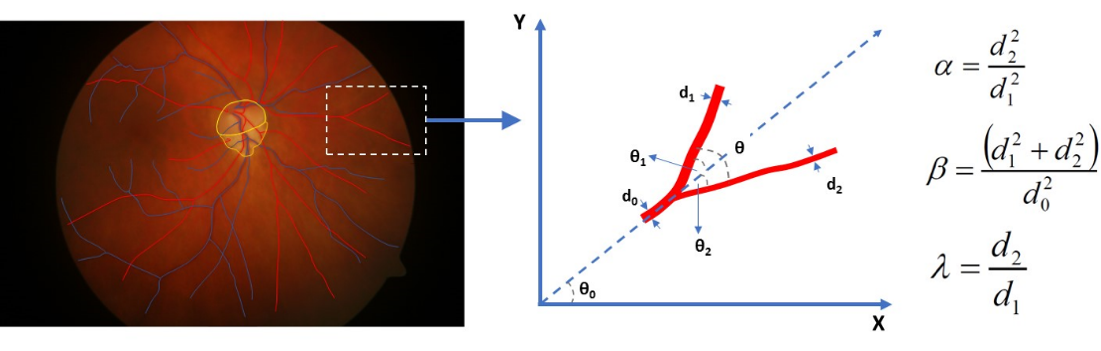


**Supplementary Figure 4.** Fundus photo with segmented retinal vessels highlighted (red – arteries, blue – veins). Features obtained from each bifurcation are shown on the right. The diameter of each vessel was measured (d0, d1 and d2). Branching geometry at the bifurcation (angle θ, θ1 and θ2) and diameter ratios (α, β and λ) are shown.
