# Supplementary material for: Optic Nerve Head and Retinal Abnormalities Associated with Congenital Fibrosis of the Extraocular Muscles
Source: Int J Mol Sci. 2021 Mar 4;22(5):2575. doi: 10.3390/ijms22052575 (PMC7961960; doi:10.3390/ijms22052575)
Supplement: Supplementary file 1 [file ijms-22-02575-s001.zip › Supplementary Table 1_revised.docx]

***Supplementary table 1: Genotype and clinical characteristics of patients with congenital fibrosis of extraocular muscles***

|  |  |  |  |  |  |  | **Corrected VA**  **(LogMAR)** | |  |  |  | **Limited Horizontal Duction** | | | | **Limited Vertical Duction** | | | | **Variant** | | | **Fundus** | |
| --- | --- | --- | --- | --- | --- | --- | --- | --- | --- | --- | --- | --- | --- | --- | --- | --- | --- | --- | --- | --- | --- | --- | --- | --- |
|  | **Affected Subject** |  |  |  |  |  |  |  |  |  |  | **RE** | | **LE** | | **RE** | | **LE** | | **Gene** | **Nucleotide change** | **Amino Acid change** | **Optic Nerve** | **Vascular** |
| **Family ID** |  | **Race** | **Gender** | **Age (years)** | **Diagnosis** | **AHP (D)** | **RE** | **LE** | **Type of Strabismus** | **Binocularity** | **Ptosis** | **Ab** | **Ad** | **Ab** | **Ad** | **Up** | **Dn** | **Up** | **Dn** |  |  |  |  |  |
| CFEOM-F001 | F1:II-3 | Asian | M | 7 | CFEOM3 | Tilt | 0.280 | 0.280 | HoT | 150" | nil | 0 | 0 | 0 | 0 | -3 | 0 | 0 | 0 | TUBB3 | c.1263G>C; heterozygous | p.E421D | Normal | Tortuous and trifurcations |
| CFEOM-F001 | F1:II-2 | Asian | M | 12 | CFEOM3 | Chin up | 0.300 | 0.220 | HoT | 150" | nil | 0 | 0 | 0 | 0 | -3 | 0 | 0 | 0 | TUBB3 | c.1263G>C; heterozygous | p.E421D | Normal | Tortuous |
| CFEOM-F001 | F1:I-1 | Asian | M | 39 | CFEOM3 | Chin up | 0.500 | 1.000 | ET | nil | Bilateral | 0 | 0 | -2 | 0 | -2 | -2 | -2 | -2 | TUBB3 | c.1263G>C; heterozygous | p.E421D | Abnormal | Bayoneting of vessels |
| CFEOM-F002 | F2:II-1 | Asian | F | 9 | CFEOM1 | Chin up and face turn | 0.275 | 0.275 | XT | nil | Bilateral | 0 | -4 | 0 | -4 | -4 | -4 | -4 | -5 | - | - | - | Abnormal | Situs inversus |
| CFEOM-F002 | F2:I-2 | Asian | F | 33 | CFEOM1 | Face turn | -0.100 | 0.500 | XT c HoT | nil | Bilateral | -0.5 | -3 | -1 | -2 | -3 | -3 | -4 | -4 | - | - | - | Normal | Normal |
| CFEOM-F003 | F3:I-2 | Caucasian | F | 65 | CFEOM1 | Chin up | 0.300 | 1.100 | XT c HoT | nil | Bilateral | -0.5 | -0.5 | -3 | -5 | -4 | -4 | -3 | -4 | KIF21A | c.2860C>T, heterozygous | p.R954W | Abnormal | Situs inversus |
| CFEOM-F003 | F3:II-2 | Caucasian | F | 40 | CFEOM1 | Nil | 1.475 | 1.770 | NA | nil | Bilateral | 0 | 0 | -4 | -4 | -3 | -3 | -4 | -4 | KIF21A | c.2860C>T, heterozygous | p.R954W | Abnormal | Situs inversus |
| CFEOM-F003 | F3:III-1 | Caucasian | F | 8 | CFEOM1 | Chin up | 0.325 | 0.500 | XT | nil | Bilateral | -0.5 | -1 | -0.5 | -1 | -4 | -4 | -3.5 | -4 | KIF21A | c.2860C>T, heterozygous | p.R954W | Pseudo-normal | Situs inversus |
| CFEOM-F004 | F4:II-1 | Caucasian | M | 12 | CFEOM3 | Chin up and face turn | 0.500 | 0.750 | XT | nil | Bilateral | 0 | -5 | 0 | -5 | -4 | -4 | -4 | -4 | TUBB3 | c.1228G>A; heterozygous | p.E410K | Pseudo-normal | Normal |
| CFEOM-F004 | F4:II-2 | Caucasian | M | 11 | CFEOM3 | Chin up and face turn | ** | ** | XT | nil | Bilateral | -2 | -5 | -1 | -5 | -4 | -4 | -4 | -4 | TUBB3 | c.1228G>A; heterozygous | p.E410K | Pseudo-normal | Normal |
| CFEOM-F004 | F4:I-2 | Caucasian | F | 35 | CFEOM3 | Chin up and face turn | 0.500 | 1.000 | XT | nil | Bilateral | -1 | -5 | -3 | -5 | -4 | -4 | -4 | -4 | TUBB3 | c.1228G>A; heterozygous | p.E410K | Pseudo-normal | Normal |
| CFEOM-F004 | F4:II-3 | Caucasian | M | 5 | CFEOM3 | Chin up and tilt | ** | ** | XT | nil | Bilateral | 0 | 0 | 0 | -3 | -4 | -4 | -4 | -4 | TUBB3 | c.1228G>A; heterozygous | p.E410K | Abnormal | Normal |
| CFEOM-S001 | S1:II-1 | Caucasian | F | 15 | CFEOM1 | Chin up, turn and tilt | 0.200 | 0.000 | NA | 85" | Bilateral | -1 | 0 | -1 | 0 | -4 | -4 | -3 | -4 | KIF21A | c.2860C>T, heterozygous | p.R954W | Abnormal | Normal |
| CFEOM-S002 | S2:II-1 | Caucasian | M | 14 | CFEOM1 | Chin up | -0.100 | 0.200 | XT c HoT | nil | Bilateral | 0 | 0 | 0 | 0 | -0.5 | -1 | -4 | -3 | TUBB3 | c.229C>T, heterozygous* | p.R77C | Normal | Normal |
| CFEOM-S003 | S3:II-1 | Caucasian | M | 11 | CFEOM3 | Chin up | 0.200 | 0.000 | XT c HoT | nil | Unilateral | 0 | -3 | 0 | 0 | -1.5 | -1.5 | 0 | 0 | - | - | - | Normal | Normal |
| CFEOM-S004 | S4:II-1 | Caucasian | F | 15 | CFEOM1 | Chin elevation | 0.440 | 0.220 | XT | nil | Bilateral | -1 | 0 | -1 | 0 | -4 | -4 | -4 | -4 | - | - | - | Pseudo-normal | Normal |

***Abbreviations:***

M = male

F = female

RE = right eye

LE = left eye

HoT = hypotropia

ET = esotropia

XT = exotropia

NA = none

Ab = abduction

Ad = adduction

Dn = down

* = Variant of unknown significance

** = not possible due to poor co-operation
