# Supplementary material for: Optic Nerve Head and Retinal Abnormalities Associated with Congenital Fibrosis of the Extraocular Muscles
Source: Int J Mol Sci. 2021 Mar 4;22(5):2575. doi: 10.3390/ijms22052575 (PMC7961960; doi:10.3390/ijms22052575)
Supplement: Supplementary file 1 [file ijms-22-02575-s001.zip › Supplementary table 2_v3.docx]

Supplementary table 2: Variants and *in silico* predictions in families and singletons with congenital fibrosis of extraocular muscles

| Family | Gene | Mutation (nt) | Mutation (aa) | MutationTaster prediction | PolyPhen2 prediction | PolyPhen2 score | CADD score | GERP score | gnomAD  Allele frequency | RS number |
| --- | --- | --- | --- | --- | --- | --- | --- | --- | --- | --- |
| F3 & S1 | *KIF21a* | c.2860C>T | p.Arg954Trp | Disease causing | Probably damaging | 0.999 | 20.600 | 3.660 | 0 | rs121912585 |
| S2 | *TUBB3* | c.229C>T | p.Arg77Cys | Disease causing | Benign | 0.176 | 12.410 | 4.570 | 0 | N/A |
| F4 | *TUBB3* | c.1228G>A | p.Glu410Lys | Disease causing | Possibly damaging | 0.813 | 15.950 | 4.660 | 0 | rs267607165 |
| F1 | *TUBB3* | c.1263G>C | p.Glu421Asp | Disease causing | Possibly damaging | 0.857 | 15.960 | 2.700 | 0 | N/A |
