# Supplementary figures and images for: Optic Nerve Head and Retinal Abnormalities Associated with Congenital Fibrosis of the Extraocular Muscles
Source: Int J Mol Sci. 2021 Mar 4;22(5):2575. doi: 10.3390/ijms22052575 (PMC7961960; doi:10.3390/ijms22052575)

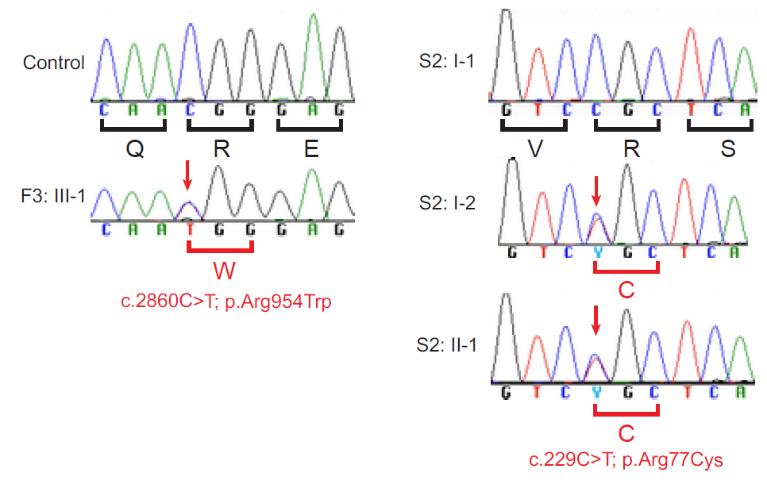


**Supplementary Figure 1.** Electropherograms from affected subjects in family F3 and family S2.

Supplement: Supplementary file 1 [file ijms-22-02575-s001.zip › Supplementary Figure S1.docx]
